# Depression, cardiometabolic disease, and their co-occurrence after childhood maltreatment: an individual participant data meta-analysis including over 200,000 participants

**DOI:** 10.1186/s12916-023-02769-y

**Published:** 2023-03-13

**Authors:** Camille Souama, Femke Lamers, Yuri Milaneschi, Christiaan H. Vinkers, Serena Defina, Linda Garvert, Frederike Stein, Tom Woofenden, Katharina Brosch, Udo Dannlowski, Henrike Galenkamp, Ron de Graaf, Vincent W. V. Jaddoe, Anja Lok, Bas B. van Rijn, Henry Völzke, Charlotte A. M. Cecil, Janine F. Felix, Hans J. Grabe, Tilo Kircher, Karim Lekadir, Margreet ten Have, Esther Walton, Brenda W. J. H. Penninx, on behalf of the EarlyCause consortium

**Affiliations:** 1 Department of Psychiatry, Amsterdam UMC, Location Vrije Universiteit Amsterdam, Boelelaan 1117, Amsterdam, The Netherlands; 2 Amsterdam Public Health, Mental Health Program, Amsterdam, The Netherlands; 3 Amsterdam Neuroscience, Mood, Anxiety, Psychosis, Stress, and Sleep Program, Amsterdam, The Netherlands; 4 Amsterdam Neuroscience, Complex Trait Genetics, Amsterdam, The Netherlands; 5 Department Anatomy & Neurosciences, Amsterdam University Medical Center Location Vrije Universiteit Amsterdam, 1081 HV Amsterdam, The Netherlands; 6 GGZ inGeest Mental Health Care, 1081 HJ Amsterdam, The Netherlands; 7 Erasmus University Medical Center Rotterdam, Department of Child and Adolescent Psychiatry/Psychology, Rotterdam, The Netherlands; 8 Generation R Study Group, Erasmus MC, University Medical Center Rotterdam, Rotterdam, The Netherlands; 9 Department of Psychiatry and Psychotherapy, University Medicine Greifswald, Ellernholzstraße 1-2, 17475 Greifswald, Germany; 10 Department of Psychiatry and Psychotherapy, Philipps-University Marburg, Marburg, Germany; 11 Department of Psychology, University of Bath, Bath, UK; 12 Institute for Translational Psychiatry, University of Münster, Münster, Germany; 13 Department of Public and Occupational Health, Amsterdam UMC, Location Academic Medical Center, University of Amsterdam, Amsterdam, The Netherlands; 14 Department of Epidemiology, Netherlands Institute of Mental Health and Addiction, Utrecht, The Netherlands; 15 Department of Paediatrics, Erasmus MC, University Medical Center Rotterdam, Rotterdam, the Netherlands; 16 Department of Psychiatry, Amsterdam UMC, Location Academic Medical Center, University of Amsterdam, Amsterdam, The Netherlands; 17 Department of Obstetrics and Fetal Medicine, Erasmus University Medical Centre, Rotterdam, The Netherlands; 18 Institute for Community Medicine, SHIP/KEF, University Medicine Greifswald, Greifswald, Germany; 19 Department of Epidemiology, Erasmus MC, University Medical Center Rotterdam, Rotterdam, The Netherlands; 20 Molecular Epidemiology, Department of Biomedical Data Sciences, Leiden University Medical Center, Leiden, The Netherlands; 21 Faculty of Mathematics and Computer Science, Artificial Intelligence in Medicine Lab, University of Barcelona, Barcelona, Spain

**Keywords:** Adverse childhood experiences, Child abuse, Childhood maltreatment, Comorbidity, Depression, Depressive disorder, Cardiovascular diseases, Diabetes mellitus, Meta-analysis

## Abstract

**Background:**

Childhood maltreatment is associated with depression and cardiometabolic disease in adulthood. However, the relationships with these two diseases have so far only been evaluated in different samples and with different methodology. Thus, it remains unknown how the effect sizes magnitudes for depression and cardiometabolic disease compare with each other and whether childhood maltreatment is especially associated with the co-occurrence (“comorbidity”) of depression and cardiometabolic disease. This pooled analysis examined the association of childhood maltreatment with depression, cardiometabolic disease, and their comorbidity in adulthood.

**Methods:**

We carried out an individual participant data meta-analysis on 13 international observational studies (*N* = 217,929). Childhood maltreatment comprised self-reports of physical, emotional, and/or sexual abuse before 18 years. Presence of depression was established with clinical interviews or validated symptom scales and presence of cardiometabolic disease with self-reported diagnoses. In included studies, binomial and multinomial logistic regressions estimated sociodemographic-adjusted associations of childhood maltreatment with depression, cardiometabolic disease, and their comorbidity. We then additionally adjusted these associations for lifestyle factors (smoking status, alcohol consumption, and physical activity). Finally, random-effects models were used to pool these estimates across studies and examined differences in associations across sex and maltreatment types.

**Results:**

Childhood maltreatment was associated with progressively higher odds of cardiometabolic disease without depression (OR [95% CI] = 1.27 [1.18; 1.37]), depression without cardiometabolic disease (OR [95% CI] = 2.68 [2.39; 3.00]), and comorbidity between both conditions (OR [95% CI] = 3.04 [2.51; 3.68]) in adulthood. Post hoc analyses showed that the association with comorbidity was stronger than with either disease alone, and the association with depression was stronger than with cardiometabolic disease. Associations remained significant after additionally adjusting for lifestyle factors, and were present in both males and females, and for all maltreatment types.

**Conclusions:**

This meta-analysis revealed that adults with a history of childhood maltreatment suffer more often from depression and cardiometabolic disease than their non-exposed peers. These adults are also three times more likely to have comorbid depression and cardiometabolic disease. Childhood maltreatment may therefore be a clinically relevant indicator connecting poor mental and somatic health. Future research should investigate the potential benefits of early intervention in individuals with a history of maltreatment on their distal mental and somatic health (PROSPERO CRD42021239288).

**Supplementary Information:**

The online version contains supplementary material available at 10.1186/s12916-023-02769-y.

## Background

Childhood maltreatment is a major public health concern [1]. Robust evidence shows that childhood maltreatment is associated with a twofold to threefold increased risk of depression in adulthood [2, 3], and this association is likely causal [4]. Depression risk is increased after experiencing any type of maltreatment, although emotional abuse and neglect seem to be particularly strong predictors [5]. The effect of childhood maltreatment on depression has also been suggested to be sex-specific, with stronger associations in females than in males [6], yet evidence is limited.

Beyond mental health, childhood maltreatment is also linked to cardiometabolic diseases in adulthood. Exposure to maltreatment in childhood is associated with an increased incidence of cardiovascular diseases (incidence rate ratio = 1.71) and type 2 diabetes (incidence rate ratio = 2.13) [7]. There seems to be a dose–response relationship; the greater the number of experienced maltreatment types, the higher the risk of cardiometabolic diseases [8]. Although this association is mostly similar across sexes, emotional neglect appears more strongly related to cardiovascular disease in females [9].

Extensive evidence shows that cardiometabolic disease and depression co-occur and are bidirectionally linked [10, 11]. Meta-analyses indicate that 29% of patients with myocardial infarction [12] and 18–32% of those with diabetes have comorbid depression [13]. Depressed individuals also have a 64–80% higher risk of developing cardiovascular disease [14, 15]. The high risk of co-occurrence, or comorbidity, is possibly explained by common underlying mechanisms. Childhood maltreatment could be a shared risk factor prompting a cascade of mechanisms leading to these diseases. In fact, depression and cardiovascular disease have a shared genetic vulnerability [16], possibly associated to biological pathways [17] such as inflammation, hypothalamic–pituitary–adrenal (HPA) axis dysregulations, and dysfunction of the autonomic nervous system; and behavioral pathways such as physical inactivity, unhealthy diet, smoking, and drinking, that may be further stimulated by childhood maltreatment, giving rise to more comorbidity between depression and cardiometabolic diseases.

Despite this evidence, no one has yet directly compared the increase in depression prevalence with the increase in cardiometabolic disease prevalence after childhood maltreatment. Investigating the associations of childhood maltreatment with depression and cardiometabolic disease in the same samples with harmonized variable definitions and a uniform handling of covariates is novel and enables the comparison of effect sizes. Additionally, although childhood maltreatment potentially activates biological and behavioral risk pathways that are shared for depression and cardiometabolic disease, no one has yet established whether childhood maltreatment is associated with the comorbidity of these diseases in adulthood. Because comorbid depression and cardiometabolic disease involve a heavier disease burden and mortality than each disease individually [18], characterizing the association with comorbidity would facilitate efforts to develop appropriate and efficient approaches to this major public health issue.

In this study, we conducted a large-scale individual participant data (IPD) meta-analysis to investigate the association of childhood maltreatment with depression, cardiometabolic disease and their comorbidity in adults. We also explored the role of childhood maltreatment type, lifestyle factors, and sex in these associations.

## Methods

### Cohorts and participants

This IPD meta-analysis is an effort of the EarlyCause consortium [19], which investigates the association between early-life stress and comorbid depression and cardiometabolic outcomes. Studies within and outside the consortium were selected based on the consortium network. Study inclusion criteria were as follows: having retrospective reports on childhood maltreatment (at least physical and emotional abuse) before the age of 18 and having data on depression and/or cardiometabolic diseases in adulthood. Studies were excluded if participants were younger than 18 years at assessment time. In total, 13 studies from Germany, the Netherlands, the UK, and the USA were included.

Three studies were case–control studies with an overrepresentation of individuals with affective disorders: the Marburg-Münster Affective Disorders Cohort Study (MACS) [20], the Netherlands Study of Depression and Anxiety (NESDA) [21], and the Netherlands Study of Depression in Older persons (NESDO) [22]. Ten studies were population-based cohort studies: the mothers and partners of the Avon Longitudinal Study of Parents and Children (ALSPAC, see Additional file 1: Sect. 1) [23–25], the mothers of Generation R Study (GenR, see Additional file 1: Sect. 2) [26], the Healthy Life in an Urban Setting (HELIUS) study [27], the first wave of the Midlife in the United States (MIDUS) study [28], the first and second Netherlands Mental Health Survey and Incidence Studies (NEMESIS-1 and NEMESIS-2) [29, 30], two independent cohorts of the Study of Health In Pomerania (SHIP-Trend baseline assessment and SHIP-Legend) [31], and the UK Biobank (UKBB) [32, 33]. Each cohort study was approved by local ethics committees and all participants provided informed consent. This research project was pre-registered on the international prospective register of systematic reviews PROSPERO in March 2021 (reference CRD42021239288). The PRISMA-IPD guidelines [34] were followed except for the systematic review-related steps which were not applicable in this research.

### Measures

#### Childhood maltreatment

The main exposure was the presence of childhood maltreatment in any of the following categories: physical, emotional, and/or sexual abuse, before the age of 18. Physical and emotional maltreatment were defined by the following: (1) self-reported history of regular or more frequent abuse (“often true”, “very often true”, “regularly”, “often”, or “very often” frequency ratings depending on the instrument) when a frequency assessment was available or (2) self-reported history of abuse in case of a dichotomous assessment. Sexual abuse was defined by the report of at least one occurrence of sexual abuse in childhood. Cases of childhood maltreatment were identified when criteria were met for either maltreatment type. Neglect was not included in the definition of childhood maltreatment because participating studies either did not assess physical and emotional neglect (*n* = 6) or assessed them in discrepant manners. Specific measures and criteria used to code childhood maltreatment (absent vs. present) in each study are described in Additional file 1: Table S1 [35–38].

#### Psycho-cardiometabolic outcomes

##### Depression

The presence of depression was defined by the following: (1) the presence of a lifetime (eight cohorts) or current (one cohort) diagnosis of major depressive disorder assessed with (semi-)structured clinical interviews or (2) current depressive symptomatology (four cohorts) assessed with self-report scales using validated clinical cut-offs. Cohort-specific measures and criteria used to identify depression cases (absent vs. present) are described in Additional file 1: Table S2 [39–48].

In sensitivity analyses, the depression definition was extended, including information on self-reported current use of antidepressants (Anatomical Therapeutic Chemical (ATC) codes starting with N06A, N06AA, N06AB, N06AF, N06AG, and N06AX) to identify additional depression cases.

##### Cardiometabolic disease

The presence of cardiometabolic disease was based on self-reports of a lifetime clinical diagnosis of a non-congenital cardiovascular disease (see Additional file 1: Table S3 for a complete list) and/or diabetes mellitus (absent vs. present). Cardiovascular disease and diabetes were selected as cardiometabolic diseases because of their known co-occurrence with depression [15, 49], their high prevalence and major impact on public health [50, 51], and their consistent assessment across cohorts.

In sensitivity analyses, we additionally tested strict (limited to heart/cardiac diseases) and broad (also including blood pressure and other heart and peripheral vascular problems; see Additional File 1: Table S3 for complete list) definitions of cardiovascular disease. Furthermore, the cardiometabolic disease definition was extended, including information on self-reported current use of related medications (ATC codes C01, C03, C04, C05, C07, C08, C09, and C10) to identify additional cases of cardiometabolic disease.

##### Comorbidity status

Comorbidity status was based on depression and cardiometabolic disease status. It comprised four levels: 0 = absence of depression and cardiometabolic disease (heathy controls), 1 = depression only, 2 = cardiometabolic disease only, 3 = comorbidity of depression and cardiometabolic disease.

In sensitivity analyses, the definition of comorbidity status was adjusted based on the strict (model 8) and broad (model 9) definitions of cardiovascular disease and on definitions of depression and cardiometabolic disease incorporating current medication use (model 10).

#### Covariates

Sociodemographic covariates sex, age, and educational attainment were assessed at the earliest timepoint available. Sex and age were based on either self-reports or municipal registries, and educational attainment was based exclusively on self-reports. Educational attainment was harmonized across cohorts and countries by using the International Standard Classification of Education (ISCED) 2011 [52] and categorized in three levels: ISCED 0–2 corresponds to no education, early childhood education, primary and lower secondary education; ISCED 3–4 corresponds to upper secondary education and post-secondary non-tertiary education; and ISCED 5–6-7–8 corresponds to short-cycle tertiary education, bachelor, master, and doctor or equivalent levels. In addition, ethnicity was entered as a sociodemographic covariate in all analyses of HELIUS due to its design-specific oversampling of participants from different ethnic groups (Dutch, Ghanaian, Moroccan, Surinamese, and Turkish). Lifestyle covariates included self-reported current smoking status, weekly alcohol consumption, and weekly physical activity. Smoking status was assessed consistently across cohorts (current smoking vs. no current smoking). Alcohol consumption and physical exercise assessments varied across cohorts and specifications are described in Additional File 1: Table S4 [53].

### Statistical analyses

A two-step IPD meta-analysis was carried out [54]. In the first step, cohorts applied a standardized protocol for data harmonization to create the required variables and carry out statistical analyses estimating the associations between childhood maltreatment and the different outcomes. In the second step, we meta-analyzed cohorts’ aggregate effect sizes with random-effects models using inverse-variance weighting. We chose random-effects models to pool the aggregate effect sizes because these effect sizes are drawn from different populations. Cohorts with cell count < 5 across exposure and outcome categories were excluded from the meta-analyses. Heterogeneity parameters *Q*, *I*^*2*^, and ${\tau }^{2}$ were calculated. Scripts of the two steps can be found on the Github EarlyCause repository (see Additional File 1: Sect. 3).

#### Main models

The main models assessed the association of childhood maltreatment with (model 1) depression (vs. no depression) and (model 2) cardiometabolic disease (vs. no cardiometabolic disease) using binomial logistic regressions and (model 3) comorbidity status (absence of disease vs. depression only vs. cardiometabolic disease only vs. comorbidity) using multinomial logistic regression. Subgroup analyses were carried out to explore whether differences in cohorts’ depression assessments possibly explained effect size heterogeneity in model 3. All models were adjusted for sociodemographic covariates. Lifestyle factors were additionally included in the model to examine their impact on the association of childhood maltreatment with comorbidity status (model 4). Analyses were then stratified by sex to check the consistency of results in males and females (models 5a and 5b). Additionally, the association of types of childhood maltreatment (physical abuse, emotional abuse, and sexual abuse) with the four-level comorbidity status was investigated in a multinomial logistic multiple regression model (model 6). Finally, we examined the role of maltreatment severity by creating a new variable, number of maltreatment types (0 type vs. 1 type vs. 2 or more types of childhood maltreatment) and testing its association with comorbidity status in a multinomial logistic regression model (model 7).

#### Sensitivity analyses

We carried out sensitivity analyses to check the consistency of the results obtained in the main model 3. First, we alternatively applied different definitions (strict and broad) of cardiovascular disease (models 8 and 9, respectively). Then, we extended the definition of depression and cardiometabolic disease incorporating information on the use of related medications (model 10).

Analyses were conducted on participants with complete data on childhood physical and emotional abuse, as well as on depression and/or cardiometabolic disease. For cohorts with 20% or more cases with missing lifestyle values in model 4 compared to the sample used in model 3, missing lifestyle values were imputed (see Additional File 1: Sect. 4 and Table S5 for detailed explanations). For cohorts with less than 20% missingness on lifestyle factors, cases with missing lifestyle values were excluded from the model. High missingness in lifestyle covariates (in particular smoking status) applied in model 4 was limited to two out of nine total cohorts. Although participants with these missing covariates represented only 2.1% of the total sample size of the pooled model 4, we decided a priori to impute lifestyle covariates when their missingness caused an important loss of data since we aimed to compare estimated associations from models with (model 4) and without lifestyle covariates (model 3). The statistical software R version 4.0.5. (packages “metafor” version 3.0–2 [55] and “meta” version 5.2–0 [56]) was used to carry out the analyses. Statistical significance level was set at *p* < 0.05, two-sided.

## Results

This study includes 13 cohorts, with a combined sample size of 217,929 participants. Weighted mean age across studies was 52.4 years. Three studies were case–control studies with a higher prevalence of depression only (weighted mean 52.4%) compared to the 10 population-based cohort studies (weighted mean 19.1%). The weighted mean prevalence of cardiometabolic disease was 5.1%, and the weighted mean prevalence of comorbidity was 2.1%. Cohort-specific information can be found in Table 1.

**Table 1:** Descriptive statistics of the participating cohorts

Cohort	Study type	*N*	Mean age (SD)	Female (%)	CM (%)	Dep. only (%)	Card. only (%)	Comorbidity dep. and card. (%)
ALSPAC, mothers	PB	3927	29.2 (4.4)	100.0	10.8	16.7	2.5	0.8
ALSPAC, partners	PB	2076	32.0 (5.1)	0.0	7.9	7.7	3.9	0.8
GenR, mothers	PB	3992	41.4 (4.5)	100.0	10.9	5.2	2.5	0.3
HELIUS	PB	20,820	44.2 (13.2)	57.5	13.1	11.5	8.5	2.4
MACS	CC	1677	35.3 (13.1)	63.8	47.4	42.3	0.5	1.6
MIDUS	PB	5988	46.7 (12.8)	52.1	20.4	9.9	11.0	2.0
NEMESIS-1	PB	7060	41.1 (12.2)	53.3	15.8	15.7	2.5	0.4
NEMESIS-2	PB	6469	44.3 (12.5)	55.2	15.4	18.3	3.6	1.3
NESDA	CC	2977	41.9 (13.1)	66.4	32.8	58.9	3.3	5.6
NESDO	CC	508	70.6 (7.3)	64.8	29.1	47.8	8.1	22.6
SHIP-Legend	PB	1882	57.2 (13.4)	53.2	12.5	7.5	22.1	3.7
SHIP-Trend	PB	4042	51.5 (15.3)	51.6	10.9	9.2	19.3	4.9
UKBB	PB	156,511	55.9 (7.7)	56.6	11.3	21.7	4.3	2.0
Total		217,929						

*Abbreviations*: *N* sample size, *CM* Childhood maltreatment, *Dep* Depression, *Card* Cardiometabolic disease, *PB* Population-based, *CC* Case-control

Age was recorded at baseline for most cohorts. Exceptions were for GenR mothers, for which age at the assessment when children were 9 years old was used; for ALSPAC partners for which age at 16 weeks of gestation was used, and for SHIP-Legend for which age at the assessment wave SHIP-Start-2 was used [31]

### Association of childhood maltreatment with depression, cardiometabolic disease, and comorbidity

**Fig. 1:**
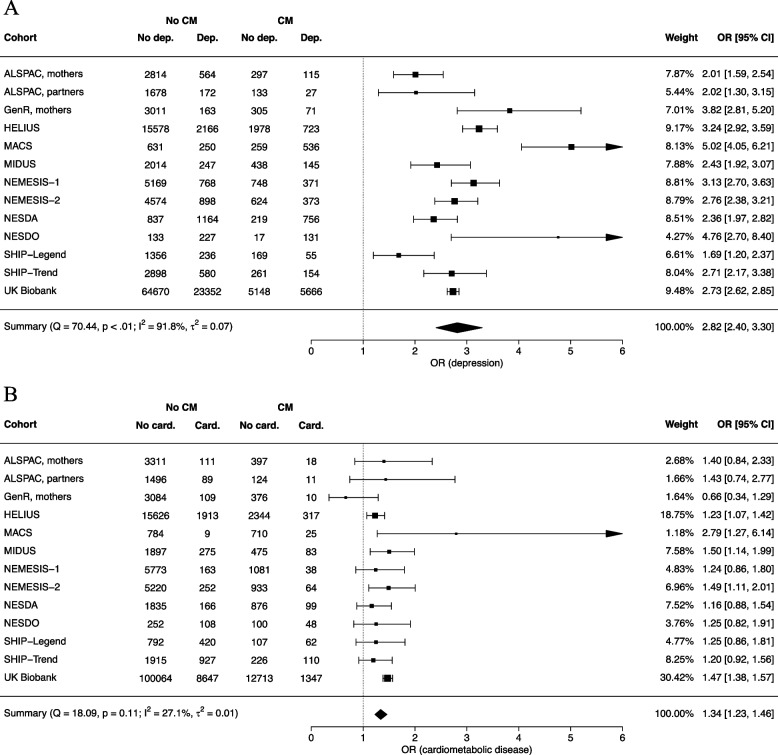
Forest plots of the random-effects models of the association of childhood maltreatment with depression (**A**) and cardiometabolic disease (**B**). CM, childhood maltreatment. Dep., depression. Card., cardiometabolic disease. OR, odds ratio. CI, confidence interval. Note. Squares represent effect sizes of individual studies. Their size reflects the precision of the estimate based on the random-effect model. The diamond represents the pooled effect size across studies in the center of the diamond, and the lower and upper 95% confidence interval limits at the left and right side of the diamond

The odds of having depression increased almost three folds in those with a history of childhood maltreatment compared to those without (model 1, OR [95% CI] = 2.82 [2.40; 3.30], Fig. 1A). This positive association was seen in all cohorts, albeit with significant heterogeneity across studies (*Q* = 70.44, *p* < .001, *I*^*2*^ = 91.8%, ${\tau }^{2}$ = 0.07). Adults with a history of childhood maltreatment, as compared to those without, were also more likely to have a cardiometabolic disease (model 2, OR [95% CI] = 1.34 [1.23; 1.46], Fig. 1B). Effect sizes were relatively homogeneous across studies (*Q* = 18.09, *p* = 0.113, *I*^*2*^ = 27.1%, ${\tau }^{2}$ = 0.01). The results of all meta-analyzed models are shown in Table 2.

**Table 2:** Overview of results of meta-analyzed models

Model specification	*k*	Outcome levels	*n*	Pooled OR [95% CI]
**Main models**				
**Binomial regressions**				
1. Association of childhood maltreatment with depression^a^	13	No depression	115,959	ref
		Depression	39,910	2.82 [2.40; 3.30]
2. Association of childhood maltreatment with cardiometabolic disease^a^	13	No cardiometabolic disease	162,511	ref
		Cardiometabolic disease	15,421	1.34 [1.23; 1.46]
2a. Outcome: diabetes mellitus^1^	12	No diabetes	168,943	ref
		Diabetes	7330	1.35 [1.19; 1.53]
2b. Outcome: cardiovascular disease^a^	11	No cardiovascular disease	165,502	ref
		Cardiovascular disease	9216	1.39 [1.26; 1.54]
**Multinomial logistic regressions**				
3. Association of childhood maltreatment with comorbidity status^a^	9	Healthy controls	99,191	ref
		Depression only	34,360	2.68 [2.39; 3.00]
		Cardiometabolic disease only	9051	1.27 [1.18; 1.37]
		Comorbidity	3565	3.04 [2.51; 3.68]
4. Model 3 + adjustment of lifestyle factors^b^	9	Healthy controls	90,166	ref
		Depression only	30,544	2.57 [2.28; 2.89]
		Cardiometabolic disease only	8181	1.25 [1.15; 1.36]
		Comorbidity	3175	2.99 [2.46; 3.63]
5a. Model 3 in males only^a^	7	Healthy controls	46,327	ref
		Depression only	10,402	2.92 [2.51; 3.40]
		Cardiometabolic disease only	5314	1.13 [1.01; 1.27]
		Comorbidity	1582	2.90 [2.37; 3.53]
5b. Model 3 in females only^a^	8	Healthy controls	49,571	ref
		Depression only	23,167	2.59 [2.30; 2.91]
		Cardiometabolic disease only	3438	1.41 [1.26; 1.57]
		Comorbidity	1942	3.49 [2.79; 4.36]
6. Model 3 with predictors^a^ = Physical abuse	6	Healthy controls	88,347^c^	ref
		Depression only	32,198^c^	1.53 [1.41; 1.66]
		Cardiometabolic disease only	8481^c^	1.45 [1.24;1.71]
		Comorbidity	3411^c^	2.06 [1.78; 2.39]
Emotional abuse		Healthy controls	88,347^c^	ref
		Depression only	32,198^c^	2.87 [2.56; 3.22]
		Cardiometabolic disease only	8481^c^	1.29 [1.11; 1.49]
		Comorbidity	3411^c^	[2.16; 3.83]
Sexual abuse		Healthy controls	88,347^c^	ref
		Depression only	32,198^c^	1.66 [1.56; 1.76]
		Cardiometabolic disease only	8481^c^	1.09 [1.01; 1.17]
		Comorbidity	3411^c^	1.60 [1.19; 2.15]
7. Model 3 with cumulation of maltreatment types as predictor^a^ = 1 type (vs. 0 type)	6	Healthy controls	88,347^c^	ref
		Depression only	32,198^c^	2.32 [2.22; 2.41]
		Cardiometabolic disease only	8482^c^	1.17 [1.07; 1.28]
		Comorbidity	3411^c^	2.37 [1.87; 3.01]
2 or more types (vs. 0 type)		Healthy controls	88,347^c^	ref
		Depression only	32,198^c^	5.14 [3.93; 6.72]
		Cardiometabolic disease only	8482^c^	1.83 [1.49; 2.26]
		Comorbidity	3411^c^	5.96 [3.59; 9.90]
**Sensitivity analyses—multinomial logistic regressions**				
8. Model 3 with outcome = comorbidity status based on strict definition of cardiovascular disease^a^	9	Healthy controls	101,203	ref
		Depression only	35,279	2.66 [2.37; 2.98]
		Cardiometabolic disease only	7030	1.28 [1.18; 1.40]
		Comorbidity	2643	3.09 [2.54; 3.75]
9. Model 3 with outcome = comorbidity status based on broad definition of cardiovascular disease^a^	11	Healthy controls	81,817	ref
		Depression only	28,612	2.84 [2.41; 3.35]
		Cardiometabolic disease only	27,178	1.11 [1.06; 1.17]
		Comorbidity	10,364	3.00 [2.69; 3.36]
10. Model 3 with outcome = comorbidity status based on medication intake in addition to reports of diagnoses^a^	7	Healthy controls	78,989	ref
		Depression only	31,266	2.83 [2.27; 3.53]
		Cardiometabolic disease only	16,896	1.13 [1.00; 1.26]
		Comorbidity	6517	2.76 [2.28; 3.35]

^a^Minimal adjustment: correction for age, sex, and education level

^b^Minimal + additional adjustment: correction for age, sex, education level, smoking status, alcohol consumption, and physical activity

^c^These sample sizes are specific to each outcome level and across all predictors and predictor levels

*Abbreviations*: *k* number of studies included, *n* sample size used in model, *OR* Odds ratio, *CI* Confidence interval, *ref* outcome reference category in logistic regressions

An overview of the cohorts included in each meta-analyzed model can be found in Additional file 1: Table S6

**Fig. 2:**
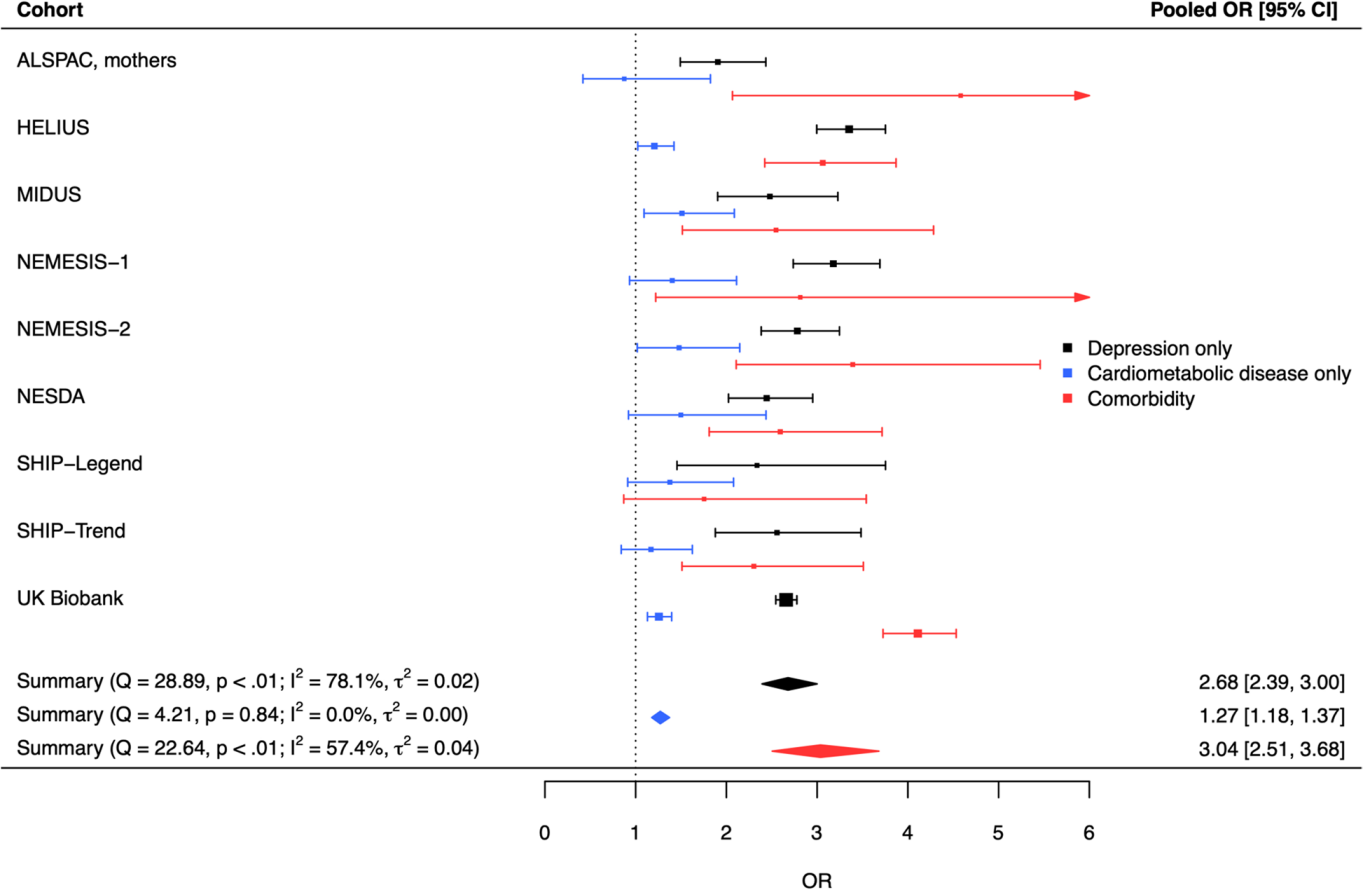
Forest plot of the random-effects model of the association of childhood maltreatment with depression only, cardiometabolic disease only, and comorbidity. Note. Number of cases, weights and odds ratios of each cohort can be found in Additional file 1: Table S7. OR: odds ratio. CI, confidence interval. Note. Squares represent effect sizes of individual studies. Their size reflects the precision of the estimate based on the random-effect model. The diamond represents the pooled effect size across studies in the center of the diamond, and the lower and upper 95% confidence interval limits at the left and right side of the diamond

The analysis of the association of childhood maltreatment with comorbidity status (model 3) was restricted to nine cohorts (*n* = 146,167) due to crosstab cells with less than five cases for some outcomes in four cohorts. In these nine cohorts, adults with a history of childhood maltreatment had twice higher odds of depression only (OR [95% CI] = 2.68 [2.39; 3.00], Fig. 2) and also higher odds of cardiometabolic disease only (OR [95% CI] = 1.27 [1.18; 1.37], Fig. 2). The effect size for cardiometabolic disease was around half of the effect size for depression only. The strongest association was found for comorbid depression and cardiometabolic disease (OR [95% CI] = 3.04 [2.51; 3.68]), Fig. 2), and this positive association was statistically significant in most cohorts. Since the UKBB represented the largest dataset in the pooled analysis (*n* = 98,619, 67.5% of participants, see Additional File 1: Table S7 for weights in pooled estimate), we re-ran the meta-analysis excluding the UKBB and observed that results remained largely similar (OR [95% CI] depression only = 2.67 [2.33; 3.06], OR [95% CI] cardiometabolic disease only = 1.29 [1.15; 1.44], OR [95% CI] comorbidity = 2.82 [2.43; 3.27]). These results were mostly consistent with results of the UKBB only (OR [95% CI] depression only = 2.66 [2.54; 2.78], OR [95% CI] cardiometabolic disease only = 1.26 [1.13; 1.40], OR [95% CI] comorbidity = 4.11 [3.73; 4.53]), although the odds of comorbidity seemed to be slightly higher in the UKBB than in the other cohorts. Subgroup analyses showed that results were largely unaffected by depression assessment type (see Additional File 1: Table S8) and by current vs. lifetime depression (see Additional File 1: Sect. 5). Additionally, we exploratively ran a post hoc test to evaluate whether childhood maltreatment was more strongly associated with comorbidity than with the individual diseases. Since the UKBB was the largest sample for which we had direct access to the individual-level data, we used that sample to calculate the following two odds ratios after childhood maltreatment: depression only vs. comorbidity and cardiometabolic disease only vs. comorbidity. Instead of using the outcome level “absence of depression and cardiometabolic disease” as reference category as in the previous calculations of odds ratios, we used the outcome level “comorbidity” as new reference category to statistically test whether childhood maltreatment was more strongly associated with comorbidity than with the single diseases. We found that the association of childhood maltreatment with depression only (OR [95% CI] = 0.65 [0.59; 0.71]) and cardiometabolic disease only (OR [95% CI] = 0.31 [0.27; 0.35]) were significantly smaller than with comorbidity.

Additional adjustment for smoking status, alcohol consumption, and physical activity (model 4) did not substantially change the associations of childhood maltreatment with depression only (OR [95% CI] = 2.57 [2.28; 2.89]), cardiometabolic disease only (OR [95% CI] = 1.25 [1.15; 1.36]), and comorbidity (OR [95% CI] = 2.99 [2.46; 3.63]), highlighting the independence of these associations from lifestyle factors. Similar associations to those obtained in the main model 3 were observed for males (model 5a) and females (model 5b). However, the association with cardiometabolic disease only seemed stronger in females than in males (OR [95% CI] in females = 1.41 [1.26; 1.57], in males = 1.13 [1.01; 1.27], with OR difference *z* = 2.77, *p* = .006; Table 2, models 5a and 5b). All types of childhood maltreatment (physical, emotional, and sexual abuse) were associated with increased odds of developing depression only, cardiometabolic disease only, and comorbidity, although physical and emotional abuse were particularly strong predictors of the comorbidity (Table 2, model 6). Finally, although all numbers of maltreatment types were related to comorbidity status, there seemed to be a dose–response relationship: When two or more types of childhood maltreatment were experienced, the odds of (comorbid) depression and cardiometabolic disease exceeded the odds found after one type of maltreatment only (Table 2, model 7).

### Sensitivity analyses

Results were similar in sensitivity analyses applied to comorbidity status model 3 when using different operational definitions of cardiovascular disease based on stricter or broader definition (models 8 and 9; Table 2) or different definitions of cardiometabolic diseases and depression additionally including information on medication (model 10; Table 2).

## Discussion

### Association of childhood maltreatment with (comorbid) depression and cardiometabolic disease

This study used data from 13 international cohorts involving 217,929 persons to systematically investigate the association of childhood maltreatment with (comorbid) depression and cardiometabolic disease in adulthood. In order to obtain a consistent set of aggregate data across cohorts, individual participant data were harmonized and cohort-level analyses were standardized. Main findings show that adults with a history of childhood maltreatment, as compared to those without, are 1.27 times more likely to have cardiometabolic disease only and 2.68 times more likely to have depression only. The largest difference between maltreated and non-maltreated individuals was found for the co-occurrence of both conditions: Maltreated individuals were 3.04 times more likely to suffer from comorbid depression and cardiometabolic disease in adulthood. Post hoc analyses showed that this association was larger than the ones for either disease alone. Results remain similar in sensitivity analyses using different outcome ascertainment definitions, suggesting findings are robust.

Our results are in line with findings from existing meta-analyses. Two relatively recent meta-analyses [2, 3] evaluated the association of childhood maltreatment history with depression and found that childhood maltreatment was associated with 2.03 (95% CI = [1.37; 3.01]) and 2.81 (95% CI = [2.35; 3.36]) increased odds of depression in adulthood, using pooled samples of 4579 [2] and 26,536 [3] participants, respectively. The current study found similar heightened odds of depression (OR [95% CI] = 2.82 [2.40; 3.30]) after childhood maltreatment, based on by far the largest sample size (*n* = 155,869). Additionally, although age was demonstrated to moderate this association [3], pooled effect sizes from the previous meta-analyses were either based on study-level effect sizes with inconsistent handling of covariates or on raw data excluding covariate adjustments. In contrast, the current research facilitated cohort-level analyses in a systematic manner, enabling the estimation of pooled effect sizes adjusting for important sociodemographic and lifestyle covariates. Although the effect size reported in Li et al. [2] is slightly smaller than the one in the current study, the difference may be explained by the definition of Li et al.’s exposure variable: Childhood maltreatment was based on official records, which are more likely to suffer from underreporting than retrospective self-reports. Despite this difference in assessment, the consistent direction of findings increase confidence in the validity of maltreatment self-reports. Further, a previous meta-analysis [57] of 29 studies (*N* = 247,393) showed that cumulative childhood adversity was moderately related to cardiometabolic disease in adulthood (OR [95% CI] = 1.36 [1.27; 1.46]). Although the exposure (an index including at least two adverse childhood experiences) and outcome (cardiometabolic disease including metabolic syndrome) definitions slightly differ from the ones of the current meta-analysis, results align closely (our findings: OR [95% CI] = 1.34 [1.23; 1.46]). Finally, because the current meta-analysis consistently adjusted associations for the same covariates, it provides the unique possibility to directly compare the increased odds of each disease after maltreatment. The results show that, compared to non-maltreated individuals, maltreated adults are 2.68 times more likely to suffer from depression and “only” 1.27 times more from cardiometabolic disease. Although linked to both, childhood maltreatment is therefore more strongly related to depression than to cardiometabolic disease in adulthood.

A striking result is that the odds of comorbid depression and cardiometabolic disease after childhood maltreatment (OR [95% CI] = 3.04 [2.51; 3.68]) are higher than for each disease alone. Although previous studies report that depression and cardiometabolic disease tend to co-occur, the current meta-analysis is the first study to investigate and support the relationship between childhood maltreatment and the co-occurrence of depression and cardiometabolic disease in adulthood. This association is possibly explained by the early-life stress triggering mechanisms common to both depression and cardiometabolic disease. Previous research suggests that childhood maltreatment activates interrelated biological and behavioral pathways [17] potentially leading to adverse health outcomes. Because childhood maltreatment occurs during a critical period for brain neuroplasticity, it may dysregulate stress-related neural circuits [58, 59]. Longitudinal studies show that childhood maltreatment is associated with structural and functional neural changes [60]. Among others, these changes may subsequently dysregulate neuroendocrine and immune systems. The HPA axis may be hyper- or hypo-activated due to impaired glucocorticoid receptor function and inflammation levels may be elevated [1, 61]. Although behavioral pathways are also hypothesized to contribute to poor health outcomes in people with childhood maltreatment [17], our results show that the associations of childhood maltreatment with comorbidity status survive adjustment for smoking, alcohol consumption and physical activity; suggesting that the increased likelihood of (comorbid) depression and cardiometabolic disease after maltreatment does not exclusively depend on one’s lifestyle. Additionally, other non-biological factors (i.e., disease severity, age at diagnosis, working conditions) may also explain the strong association observed between childhood maltreatment and comorbidity and should be investigated. Lastly, the higher odds of comorbidity than single diseases after childhood maltreatment may be explained by the fact that depression and cardiometabolic disease have a bidirectional feedforward loop [10]. Both diseases likely magnify each other in a reinforcing vicious cycle, which is further stimulated by childhood maltreatment and its related biological, psychosocial and behavioral consequences.

### Differential effects of sex and maltreatment types

We carried out additional analyses to explore how the associations between childhood maltreatment and (comorbid) depression and cardiometabolic disease varied across sex and maltreatment types. Both in males and females, childhood maltreatment was associated with more (comorbid) depression and cardiometabolic disease. Associations between childhood maltreatment and comorbidity status were mostly similar across males and females. However, females showed a slightly stronger association than males for cardiometabolic disease only (males: OR [95% CI] = 1.13 [1.01; 1.27], females: OR [95% CI] = 1.41 [1.26; 1.57]). Evidence from the literature on that matter is inconsistent [9, 57], and conclusions should therefore be drawn carefully. Further analyses were carried out to test the relationship between maltreatment types and (comorbid) depression and cardiometabolic disease. Because multi-type maltreatment is common [62], the different maltreatment types were entered as multiple predictors within the same model to obtain average estimates of the association between each maltreatment type with comorbidity status while accounting for the co-occurring experience of other types of maltreatment. Findings show that all maltreatment types were independently associated with (comorbid) depression and cardiometabolic disease. Zooming in on specific outcomes, depression only was particularly strongly associated with emotional abuse. Cardiometabolic disease only and comorbidity were particularly strongly associated with physical and emotional abuse. Previous research findings support our results: Physical and emotional abuse are stronger predictors of depression and cardiovascular disease than sexual abuse [3, 9]. Alternatively, the estimated associations of sexual abuse with the disease outcomes may be harder to detect because of the relatively low prevalence of sexual abuse compared to the other types of abuse [63] or because milder forms of sexual abuse are picked up, for instance from the population-based studies. Lastly, findings endorse a dose–response relationship of childhood maltreatment severity—here operationalized as the number of maltreatment types—with (comorbid) depression and cardiometabolic disease. This converges with previous evidence on various health outcomes [64, 65].

### Strengths and limitations

This study has several strengths. First, the meta-analysis gathered 13 international cohorts including 217,929 individuals from European countries and the USA. Second, the systematic methodology used with the two-step individual participant data design has essential advantages [54]. It enables the standardization of analyses across studies (i.e., harmonization of variables and consistent covariate adjustment of estimates) and increases the quality of aggregate data entering the meta-analysis. Third, the variety of cohorts involved (e.g., case–control and population-based studies; cohort oversampling persons with migration background) and comprehensiveness of the analyses carried out (e.g., sensitivity analyses with different outcome definitions, effects of different maltreatment types, stratified analyses across sex) strengthens the robustness of the findings across settings.

This study also has limitations. In some cohorts, especially those with younger samples, the prevalence of comorbid depression and cardiometabolic disease was low (weighted mean 2.1%), leading to some studies being excluded of the multinomial regression analyses due to small cell count. This is likely because cardiometabolic events usually happen in later life [66]. The prevalence of comorbidity increases with age as seen in the oldest cohort NESDO with the highest rate of comorbidity (22.6%). The relatively high average age across cohorts (52.4 years old) may have thereby facilitated finding existing associations. Despite the difference in outcome prevalence across cohorts of different ages, the associations found for depression and cardiometabolic disease are consistent across younger (e.g., ALSPAC mothers and partners) and older (e.g., NESDO and SHIP-Legend) cohorts. Therefore, there is no obvious indication of a differential effect of age. An alternative explanation for the low prevalence of comorbidity may be survival bias where patients with severe depression and cardiometabolic disease have died or are too ill to participate in the studies. Nevertheless, even after excluding studies with too few comorbidity cases from the multinomial regression analyses, the total sample used to investigate the association with comorbidity status still amounted to 146,167 individuals. Another limitation is that meta-analyzed associations of childhood maltreatment with depression and comorbidity showed non-negligible heterogeneity. However, we used random-effect models which, by definition, assume the included studies have different true effect sizes, and thereby account for heterogeneity in calculating pooled estimates. The heterogeneity could not be explained by differences in depression assessment but other factors could have possibly caused this divergence (e.g., study design, age, cultural differences in stigma reporting childhood maltreatment) and should be further investigated. Additionally, as with every assessment type, the reliance on self-reports has its set of limitations. Cardiometabolic diseases were assessed with self-reported diagnoses, which may be perceived as biased. However, previous research show that cardiometabolic disease assessment (self-reports vs. medical records) does not influence the association found between childhood maltreatment and cardiometabolic disease [9]. Childhood maltreatment was also assessed with self-reports. It has been suggested that depression may negatively bias someone’s recall of their childhood experiences [67] in which case, self-reports may spuriously inflate the association found between childhood maltreatment and depression as well as comorbidity. Recent evidence from published and unpublished research [67, 68] highlights the marginal susceptibility of maltreatment self-reports to negative recall bias as well as their temporal stability irrespective of depression diagnosis. In order to test this in the current study, we compared the associations found in population-based cohorts using lifetime vs. current depression assessments (see Additional file 1: Sect. 5) and found no evidence of negative recall bias. In addition, analyses were exclusively carried out on individuals with available data on childhood maltreatment. This may have introduced some bias as maltreatment non-response might be associated with the disease outcomes [8]. Moreover, the current study’s assessment of maltreatment was limited to experiences of abuse because neglect was assessed so differently across cohorts that we could not harmonize. Yet, childhood neglect is an important early-life stressor potentially affecting depression and cardiometabolic outcomes in adulthood and should be investigated in future studies. Furthermore, although the current study focusses on the comorbidity of depression with cardiovascular disease and diabetes, other comorbid psychiatric and somatic diseases may also be activated by early-life stress pathways and warrant further investigation. Another limitation concerns the fact that the current study did not test the role of maltreatment timing. Although a recent meta-analysis shows no evidence of consistent sensitive periods of childhood maltreatment linked to various health outcomes [69], future studies with detailed timing information are needed to determine with more certainty whether timing of childhood maltreatment exposure matters for (comorbid) depression and cardiometabolic disease. Finally, a last limitation concerns the unknown timeline of events. Although depression and cardiometabolic disease likely have their onset after—and we believe are caused by mechanisms stemming from—childhood maltreatment, the current study only articulates associations and does not inform about causality.

### Implications

The current findings have clinical implications. First, our results raise awareness on the association between early-life stress and distal psychiatric and somatic health. Second, this study may be a first step in the process of preserving the health of individuals with a history of childhood maltreatment. If future evidence supports that childhood maltreatment triggers a cascade of mechanisms leading to (comorbid) depression and cardiometabolic disease, early intervention could prevent the dysregulation of biological stress systems and preserve the health of individuals with a history of childhood maltreatment. For instance, standard psychotherapy has been shown to effectively reduce depression severity in individuals with a history of childhood maltreatment [70]. One could therefore consider providing trauma-focused psychotherapy to help victims of maltreatment process the stress evoked by the trauma, or pharmacotherapy aiming to regulate biological stress systems, subsequently promoting somatic and mental health. In addition to individual interventions, societal action is an opportunity to prevent these comorbid diseases. Recent influential work emphasizes that promoting fair distribution of income, protecting work conditions, fostering gender equity, decreasing discrimination, and improving social cohesion/support have a great potential to prevent early-life stress, and in turn (comorbid) depression and cardiometabolic disease [71, 72].

## Conclusions

In sum, adults with a history of childhood maltreatment are more likely to suffer from depression and cardiometabolic disease than those without a history of childhood maltreatment. Notably, childhood maltreatment is more strongly associated with the comorbidity of the two diseases than with each disease alone suggesting shared mechanisms. Since childhood maltreatment appears to be a relevant indicator linking poor mental and somatic adult health, the findings emphasize the need for the fields of pediatrics, psychiatry, cardiology, and endocrinology to collaborate in efforts to improve health outcomes.

## Supplementary Information

**Additional file 1. ** **Section 1**. ALSPAC participants. **Section 2**. GenR additional information. **Section 3.** R-script of analyses. **Section 4.** Imputation of lifestyle variables. **Section 5.** Associations with current vs. lifetime depression diagnoses. **TableS1.** Childhood maltreatment assessment overview. **Table S2.** Depressionassessment overview. **Table S3.** Definition of cardiovascular disease. **Table S4.** Alcohol consumption and physical activity assessment overview. **Table S5.** Pooled associations of childhood maltreatment with comorbidity status after adjusting for lifestyle factors (model 4), according to three different imputation strategies. **Table S6.** Overview of cohorts included in each meta-analyzed model. **Table S7.** Number of cases, weights and odds ratios of thecohorts in meta-analyzed model 3. **Table S8.** Results of meta-analyzed model 3 per subgroup of studies based on depression assessment type.

## Data Availability

The code used to carry out statistical analyses and extract aggregate data from each of the included cohort studies, as well as to synthesize the aggregate data are available on the GitHub repository of EarlyCause. It can be accessed from the EarlyCause portal (portal.earlycause.eu/tools) or directly from the GitHub repository (github.com/camillesouama/earlycause-tools/tree/main/Amsterdam%20UMC/Meta-analysis%20on%20childhood%20maltreatment%20and%20(comorbid)%20depression%20and%20cardiometabolic%20disease). Individual participant data from the included cohorts are available from management teams of ALSPAC, GenR, HELIUS, MACS, NESDA, NESDO, NEMESIS, SHIP, and UKBB but restrictions apply to the availability of these data, which were used under license for the current study and are not publicly available. For ALSPAC specifically, please note that the study website contains details of all the data that is available through a fully searchable data dictionary and variable search tool: http://www.bristol.ac.uk/alspac/researchers/our-data/. Derived aggregate data are available from the authors upon reasonable request and with permission of the management teams of ALSPAC, GenR, HELIUS, MACS, NESDA, NESDO, NEMESIS, SHIP, and UKBB. Individual participant data from MIDUS is publicly available (28).

## Abbreviations

ALSPAC: Avon Longitudinal Study of Parents And Children
ATC: Anatomical therapeutic chemical
Card.: Cardiometabolic disease
CC: Case–control
CI: Confidence interval
CM: Childhood maltreatment
Dep.: Depression
GenR: Generation R
HELIUS: Healthy Life In Urban Setting
HPA: Hypothalamic–pituitary–adrenal
IPD: Individual participant data
ISCED: International Standard Classification of Education
k: Number of studies
MACS: Marburg-Münster Affective Disorders Cohort Study
MIDUS: Midlife in the United States
N: Sample size
NEMESIS: Netherlands Mental Health Survey and Incidence Study
NESDA: Netherlands Study of Depression and Anxiety
NESDO: Netherlands Study of Depression in Older Persons
OR: Odds ratio
PB: Population-based
PRISMA-IPD: Preferred reporting items for a systematic review and meta-analysis of individual participant data
ref: Reference category
SHIP: Study of Health in Pomerania
UK: United Kingdom
UKBB: UK Biobank
USA: United States of America
