# Supplementary material for: Depression, cardiometabolic disease, and their co-occurrence after childhood maltreatment: an individual participant data meta-analysis including over 200,000 participants
Source: BMC Med. 2023 Mar 13;21:93. doi: 10.1186/s12916-023-02769-y (PMC10010035; doi:10.1186/s12916-023-02769-y)
Supplement: Supplementary file 1 — Additional file 1. Section 1. ALSPAC participants. Section 2. GenR additional information. Section 3. R-script of analyses. Section 4. Imputation of lifestyle variables. Section 5. Associations with current vs. lifetime depression diagnoses. TableS1. Childhood maltreatment assessment overview. Table S2. Depressionassessment overview. Table S3. Definition of cardiovascular disease. Table S4. Alcohol consumption and physical activity assessment overview. Table S5. Pooled associations of childhood maltreatment with comorbidity status after adjusting for lifestyle factors (model 4), according to three different imputation strategies. Table S6. Overview of cohorts included in each meta-analyzed model. Table S7. Number of cases, weights and odds ratios of thecohorts in meta-analyzed model 3. Table S8. Results of meta-analyzed model 3 per subgroup of studies based on depression assessment type. [file 12916_2023_2769_MOESM1_ESM.docx]

**Additional File 1**

Table of Contents

[1. Supplemental methods Sections 2](#_Toc121840515)

[Section 1, ALSPAC participants 2](#_Toc121840516)

[Section 2, GenR additional information 3](#_Toc121840517)

[Section 3, R-script of analyses 4](#_Toc121840518)

[Section 4, imputation of lifestyle variables 5](#_Toc121840519)

[Section 5, associations with current vs. lifetime depression diagnoses 6](#_Toc121840520)

[2. Supplemental Tables 7](#_Toc121840521)

[Table S1. *Childhood maltreatment assessment overview.* 7](#_Toc121840522)

[Table S2. *Depression assessment overview.* 8](#_Toc121840523)

[Table S3. *Definition of cardiovascular disease.* 9](#_Toc121840524)

[Table S4. *Alcohol consumption and physical activity assessment overview.* 10](#_Toc121840525)

[Table S5. *Pooled associations of childhood maltreatment with comorbidity status after adjusting for lifestyle factors (model 4), according to three different imputation strategies.* 11](#_Toc121840526)

[Table S6. *Overview of cohorts included in each meta-analyzed model.* 12](#_Toc121840527)

[Table S7. *Number of cases, weights and odds ratios of the cohorts in meta-analyzed model 3.* 13](#_Toc121840528)

[Table S8. *Results of meta-analyzed model 3 per subgroup of studies based on depression assessment type.* 14](#_Toc121840529)

1. **Supplemental Methods Sections**

## Section 1, ALSPAC participants

Pregnant women resident in Avon, UK with expected dates of delivery 1st April 1991 to 31st December 1992 were invited to take part in the study. The initial number of pregnancies enrolled is 14,541 (for these at least one questionnaire has been returned or a “Children in Focus” clinic had been attended by 19/07/99). Of these initial pregnancies, there was a total of 14,676 fetuses, resulting in 14,062 live births and 13,988 children who were alive at 1 year of age. When the oldest children were approximately 7 years of age, an attempt was made to bolster the initial sample with eligible cases who had failed to join the study originally. As a result, when considering variables collected from the age of seven onwards (and potentially abstracted from obstetric notes) there are data available for more than the 14,541 pregnancies mentioned above. The number of new pregnancies not in the initial sample (known as Phase I enrolment) that are currently represented on the built files and reflecting enrolment status at the age of 24 is 913 (456, 262 and 195 recruited during Phases II, III and IV respectively), resulting in an additional 913 children being enrolled. The phases of enrolment are described in more detail in the cohort profile paper and its update (see footnote 4 below). The total sample size for analyses using any data collected after the age of seven is therefore 15,454 pregnancies, resulting in 15,589 fetuses. Of these 14,901 were alive at 1 year of age.

## Section 2, GenR additional information

GenR is conducted by the Erasmus MC, University Medical Center Rotterdam in close collaboration with the School of Law and Faculty of Social Sciences of Erasmus University Rotterdam, the Municipal Health Service Rotterdam area, Rotterdam, the Rotterdam Homecare Foundation, Rotterdam and the Stichting Trombosedienst & Artsenlaboratorium Rijnmond (STAR-MDC), Rotterdam.

## Section 3, R-script of analyses

The R-script of the analyses can be found on the EarlyCause GitHub repository. It can be accessed from the EarlyCause portal ([portal.earlycause.eu/tools](https://portal.earlycause.eu/tools)) or directly from the GitHub repository ([github.com/camillesouama/earlycause-tools/tree/main/Amsterdam%20UMC/Meta-analysis%20on%20childhood%20maltreatment%20and%20(comorbid)%20depression%20and%20cardiometabolic%20disease](http://github.com/camillesouama/earlycause-tools/tree/main/Amsterdam%20UMC/Meta-analysis%20on%20childhood%20maltreatment%20and%20(comorbid)%20depression%20and%20cardiometabolic%20disease)).

## Section 4, Imputation of lifestyle variables

When adding lifestyle variables (model 4) to the main multinomial model (model 3), missing lifestyle variables were imputed for each cohort where the sample size decreased by 20% or more from model 3 to model 4. Missing lifestyle variables in ALSPAC mothers (24.3% for smoking, 0.5% for alcohol consumption, and 10.8% for physical activity) and in NEMESIS-1 (21.4% for smoking, < 0.01% for alcohol consumption, and < 0.01% for physical activity) were imputed with multiple imputation via predictive mean matching in the ‘mice’ R package, using 60 iterations and 30 imputations.

In order to evaluate the impact of the imputation decision on the overall results, we meta-analyzed model 4 adjusting for lifestyle factors in three different manners – once excluding cohorts with 20% or more missingness on lifestyle, once including all cohorts but without imputing those with 20% or more missingness on lifestyle, and once including all cohorts and imputing those with 20% or more missingness on lifestyle – to check whether the results changed with imputation. As displayed in supplemental table S5, the results of model 4 obtained with the three different strategies were substantially similar, suggesting that the criteria and strategies adopted for imputation had limited impact on the main results.

## Section 5, Associations with current vs. lifetime depression diagnoses

In order to test whether individuals with current depression recall the past in a more negative light than individuals with non-current depression, we compared the associations of childhood maltreatment with depression in population-based cohorts using current vs. lifetime depression assessments. Even though cases of lifetime depression may include cases of current depression, if negative recall plays a major role in maltreatment self-reports we would expect the relationship between maltreatment and depression to be stronger if it is current than if it is lifetime. By running this analysis in population-based cohorts only, we avoid the greater overlap expected in case-control studies between current and lifetime depression cases. Population-based cohorts that assessed lifetime depression were NEMESIS-1, NEMESIS-2, SHIP-Legend, SHIP-Trend, and UKBB, and those that assessed current depression were ALSPAC mothers, ALSPAC partners, GenR mothers, HELIUS, and MIDUS. The pooled association between childhood maltreatment and depression among these cohorts assessing lifetime depression was OR [95%CI]=2.70 [2.39;3.05], and among those assessing current depression was OR [95%CI]=2.66 [2.08;3.39]. Both association estimates are very similar, implying that childhood maltreatment is similarly associated to depression, regardless of current or lifetime diagnosis. Additionally, we carried out a meta-regression that shows that the association of maltreatment with depression does not significantly differ across those population-based cohorts that use current vs. lifetime assessments of depression (*β*=-0.01, *SE*=0.15, *p*=.921). This suggests that recency of depression during childhood maltreatment assessment does not play a major role in the current study findings.

1. **Supplemental Tables**

| **Cohort** | **Instrument** | **Response type** | **Definition  physical abuse** | **Definition emotional abuse** | **Definition sexual abuse** |
| --- | --- | --- | --- | --- | --- |
| ALSPAC, mothers | Single item | D | SR yes | SR yes | SR yes |
| ALSPAC, partners | Single item | D | SR yes | SR yes | SR yes |
| GenR, mothers | CTQ | LSF | SR at least regularly | SR at least regularly | SR at least once |
| HELIUS | CTI | LSF | SR at least regularly | SR at least regularly | SR at least once |
| MACS | CTQ | LSF | SR at least regularly | SR at least regularly | SR at least once |
| MIDUS | Conflict Tactics Inventory | LSF | SR at least regularly | SR at least regularly | Not assessed |
| NESDA | CTI | LSF | SR at least regularly | SR at least regularly | SR at least once |
| NESDO | CTI | LSF | SR at least regularly | SR at least regularly | SR at least once |
| NEMESIS-1 | CTI | LSF | SR at least regularly | SR at least regularly | SR at least once |
| NEMESIS-2 | CTI | LSF | SR at least regularly | SR at least regularly | SR at least once |
| SHIP-Legend | CTQ | LSF | SR at least regularly | SR at least regularly | SR at least once |
| SHIP-Trend | CTQ | LSF | SR at least regularly | SR at least regularly | SR at least once |
| UKBB | Childhood Trauma Screener | LSF | SR at least regularly | SR at least regularly | SR at least once |

## Table S1. *Childhood maltreatment assessment overview.*

*Abbreviations*. CTQ: Childhood Trauma Questionnaire. CTI: Childhood Trauma Inventory. D: dichotomous. LSF: Likert-type scale of frequency. SR: self-reported.

| **Cohort** | **Instrument** | **Instrument type** | **Definition of depression** |
| --- | --- | --- | --- |
| ALSPAC, mothers | EPDS | SR depressive symptomatology scale | Cut-off ≥ 13 of probable depression in past seven days |
| ALSPAC, partners | EPDS | SR depressive symptomatology scale | Cut-off ≥ 13 of probable depression in past seven days |
| GenR, mothers | BSI | SR depressive symptomatology scale | Cut-off ≥ 0.75 of probable depression in past seven days |
| HELIUS | PHQ-9 | SR depressive symptomatology scale | Cut-off ≥ 10 of probable depression in past two weeks |
| MACS | SCID-I | Clinical interview | Lifetime diagnosis |
| MIDUS | CIDI short form | Clinical interview | Current diagnosis |
| NESDA | CIDI, version 2.1 | Clinical interview | Lifetime diagnosis |
| NESDO | CIDI, version 2.1 | Clinical interview | Lifetime diagnosis |
| NEMESIS-1 | CIDI, version 1.1 | Clinical interview | Lifetime diagnosis |
| NEMESIS-2 | CIDI, version 3.0 | Clinical interview | Lifetime diagnosis |
| SHIP-Legend | Munich-CIDI | Clinical interview | Lifetime diagnosis |
| SHIP-Trend | Munich-CIDI | Clinical interview | Lifetime diagnosis |
| UKBB | CIDI short form | Clinical interview | Lifetime diagnosis, determined by algorithm |

## Table S2. *Depression assessment overview.*

*Abbreviations*. EPDS: Edinburgh Postnatal Depression Scale Childhood Trauma. BSI: Brief Symptom Inventory. PHQ-9: Patient Health Questionnaire 9. SCID-I: Structured Clinical Interview for Axis-1 disorders. CIDI: Composite International Diagnostics Interview. SR: self-report.

| **Definition** | **Diseases included** |
| --- | --- |
| Main | Coronary heart disease, myocardial infarction/heart attack, angina pectoris, heart failure/enlarge heart, arrhythmia/atrial fibrillation, heart valve problem/aortic insufficiency/aortic regurgitation, hole in heart/atrial or ventricular septal defect, heart murmur, cardiomyopathy, stroke, transient ischemic attack, aortic aneurysm, atherosclerosis/narrowing or hardening of arteries/claudication, pulmonary embolism/blood clot, deep vein thrombosis, phlebitis, chronic venous insufficiency, angioplasty |
| Strict | Coronary heart disease, myocardial infarction/heart attack, angina pectoris, heart failure/enlarge heart, arrhythmia/atrial fibrillation, heart valve problem/aortic insufficiency/aortic regurgitation, hole in heart/atrial or ventricular septal defect, heart murmur, cardiomyopathy |
| Broad | Coronary heart disease, myocardial infarction/heart attack, angina pectoris, heart failure/enlarge heart, arrhythmia/atrial fibrillation, heart valve problem/aortic insufficiency/aortic regurgitation, hole in heart/atrial or ventricular septal defect, heart murmur, cardiomyopathy, stroke, transient ischemic attack, aortic aneurysm, atherosclerosis/narrowing or hardening of arteries/claudication, pulmonary embolism/blood clot, deep vein thrombosis, phlebitis, chronic venous insufficiency, angioplasty, blood pressure anomalies, varicose veins and non-specified cardiovascular disease |

## Table S3. *Definition of cardiovascular disease.*

| **Cohort** | **Alcohol consumption definition** | **Physical activity definition** |
| --- | --- | --- |
| ALSPAC, mothers | Weekly frequency of alcohol consumption | Hours per week engaging in physical activity |
| ALSPAC, partners | Number of alcoholic drinks drank per week | Hours per week engaging in physical activity |
| GenR, mothers | Number of alcoholic drinks drank per week | Hours per week engaging in physical activity |
| HELIUS | Number of alcoholic drinks drank per week | Hours per week engaging in physical activity |
| MACS | Number of alcoholic drinks drank per week |  |
| MIDUS | Number of alcoholic drinks drank per week in year one drank most | Hours per week engaging in moderate and vigorous physical activity |
| NESDA | Number of alcoholic drinks drank per week | Hours per week engaging in physical activity |
| NESDO | Number of alcoholic drinks drank per week | Hours per week engaging in physical activity |
| NEMESIS-1 | Number of alcoholic drinks drank per week in the past year | Hours per week engaging in physical activity |
| NEMESIS-2 | Number of alcoholic drinks drank per week in past year | Dichotomous variable based on Dutch recommendation for weekly number of 30-minute sessions of moderate or vigorous physical activity: less than five days per week vs. five days or more per week |
| SHIP-Legend | Number of alcoholic drinks drank per week | Hours per week spent working out |
| SHIP-Trend | Number of alcoholic drinks drank per week | Hours per week spent working out |
| UKBB | Number of alcoholic drinks drank per week | Hours per week engaging in physical activity |

## Table S4. *Alcohol consumption and physical activity assessment overview.*

## Table S5. *Pooled associations of childhood maltreatment with comorbidity status after adjusting for lifestyle factors (model 4), according to three different imputation strategies.*

|  | Pooled associations excluding cohorts with 20% or more missingness on lifestyle covariates | Pooled associations including cohorts with 20% or more missingness on lifestyle covariates, without imputation | Pooled associations including cohorts with 20% or more missingness on lifestyle covariates, with imputation |
| --- | --- | --- | --- |
| **Outcome** | **OR [95% CI]** | **OR [95% CI]** | **OR [95% CI]** |
| Depression only | 2.61 [2.33; 2.93] | 2.56 [2.28; 2.88] | 2.57 [2.28; 2.89] |
| Cardiometabolic disease only | 1.25 [1.15; 1.36] | 1.24 [1.15; 1.35] | 1.25 [1.15; 1.36] |
| Comorbidity | 2.90 [2.34; 3.58] | 2.89 [2.35; 3.55] | 2.99 [2.46; 3.63] |

*Note*. OR = odds ratio. CI = confidence interval

## Table S6. *Overview of cohorts included in each meta-analyzed model.*

| **Cohort** | **1** | **2** | **2a** | **2b** | **3** | **4** | **5a** | **5b** | **6** | **7** | **8** | **9** | **10** |
| --- | --- | --- | --- | --- | --- | --- | --- | --- | --- | --- | --- | --- | --- |
| ALSPAC, mothers | x | x | x | x | x | x |  | x |  |  | x | x |  |
| ALSPAC, partners | x | x | x |  |  |  |  |  |  |  |  |  |  |
| GenR, mothers | x | x |  | x |  |  |  |  |  |  |  |  |  |
| HELIUS | x | x | x | x | x | x | x | x | x | x | x | x | x |
| MACS | x | x | x |  |  |  |  |  |  |  |  | x | x |
| MIDUS | x | x | x | x | x | x | x | x |  |  | x | x | x |
| NEMESIS-1 | x | x | x | x | x | x | x |  |  |  | x | x |  |
| NEMESIS-2 | x | x | x | x | x | x | x | x | x | x | x | x |  |
| NESDA | x | x | x | x | x | x | x | x | x | x | x | x | x |
| NESDO | x | x | x | x |  |  |  |  |  |  |  | x |  |
| SHIP-Legend | x | x | x | x | x | x |  | x | x | x | x | x | x |
| SHIP-Trend | x | x | x | x | x | x | x | x | x | x | x | x | x |
| UKBB | x | x | x | x | x | x | x | x | x | x | x | x | x |

*Note*. x = cohort included in meta-analyzed model. Most exclusions of cohorts from models are due to a cell count <5 in the crosstab of maltreatment exposure by outcome levels. Exceptions are: ALSPAC, mothers and partners were not included in model 10 because of absence of data on medication intake. ALSPAC, partners were not included in model 9 because of absence of data on broad definition of cardiovascular disease. MACS was not included in model 4 because of absence of data on physical activity. MIDUS was not included in models 6 and 7 because of absence of data on sexual abuse. NEMESIS-1 and -2 were not included in model 10 because of absence of data on antidepressant or cardiometabolic-related medication.

## Table S7. *Number of cases, weights and odds ratios of the cohorts in meta-analyzed model 3.*

| **Outcome level** | **Cohort** | **No CM (n)** | **CM (n)** | **Weight** | **OR [95% CI]** |
| --- | --- | --- | --- | --- | --- |
| Healthy controls | ALSPAC, mothers | 2,710 | 287 |  | ref. |
|  | HELIUS | 13,812 | 1,742 |  | ref. |
|  | MIDUS | 1,703 | 362 |  | ref. |
|  | NEMESIS-1 | 5,025 | 718 |  | ref. |
|  | NEMESIS-2 | 4,380 | 587 |  | ref. |
|  | NESDA | 768 | 191 |  | ref. |
|  | SHIP-Legend | 679 | 75 |  | ref. |
|  | SHIP-Trend | 1,581 | 146 |  | ref. |
|  | UK Biobank | 59,706 | 4,719 |  | ref. |
| Depression only | ALSPAC, mothers | 535 | 104 | 9.6% | 1.91 [1.49; 2.43] |
|  | HELIUS | 1,757 | 598 | 14.8% | 3.35 [3.00; 3.75] |
|  | MIDUS | 194 | 113 | 8.9% | 2.48 [1.90; 3.23] |
|  | NEMESIS-1 | 748 | 363 | 13.3% | 3.18 [2.74; 3.69] |
|  | NEMESIS-2 | 840 | 346 | 13.1% | 2.78 [2.38; 3.25] |
|  | NESDA | 1067 | 685 | 11.7% | 2.44 [2.02; 2.95] |
|  | SHIP-Legend | 110 | 31 | 4.3% | 2.34 [1.46; 3.75] |
|  | SHIP-Trend | 294 | 74 | 7.5% | 2.56 [1.88; 3.48] |
|  | UK Biobank | 21,445 | 5,056 | 16.9% | 2.66 [2.54; 2.78] |
| Cardiometabolic disease only | ALSPAC, mothers | 89 | 8 | 1.1% | 0.87 [0.42; 1.82] |
|  | HELIUS | 1,527 | 208 | 21.7% | 1.21 [1.02; 1.42] |
|  | MIDUS | 230 | 59 | 5.6% | 1.51 [1.09; 2.09] |
|  | NEMESIS-1 | 144 | 30 | 3.5% | 1.40 [0.93; 2.11] |
|  | NEMESIS-2 | 194 | 37 | 4.2% | 1.48 [1.02; 2.15] |
|  | NESDA | 69 | 28 | 2.5% | 1.50 [0.92; 2.44] |
|  | SHIP-Legend | 357 | 50 | 3.5% | 1.38 [0.91; 2.08] |
|  | SHIP-Trend | 705 | 65 | 5.4% | 1.17 [0.84; 1.62] |
|  | UK Biobank | 4,836 | 415 | 52.4% | 1.26 [1.13; 1.40] |
| Comorbidity | ALSPAC, mothers | 21 | 9 | 4.7% | 4.58 [2.07; 10.16] |
|  | HELIUS | 371 | 109 | 18.2% | 3.06 [2.42; 3.87] |
|  | MIDUS | 45 | 24 | 8.8% | 2.55 [1.51; 4.28] |
|  | NEMESIS-1 | 19 | 8 | 4.4% | 2.81 [1.22; 6.49] |
|  | NEMESIS-2 | 58 | 27 | 9.9% | 3.39 [2.11; 5.46] |
|  | NESDA | 97 | 71 | 13.3% | 2.59 [1.81; 3.72] |
|  | SHIP-Legend | 57 | 11 | 5.8% | 1.75 [0.87; 3.54] |
|  | SHIP-Trend | 163 | 33 | 11.4% | 2.30 [1.51; 3.51] |
|  | UK Biobank | 1,850 | 592 | 23.5% | 4.11 [3.73; 4.53] |

*Abbreviations.* CM = childhood maltreatment. OR = Odds ratio. CI = confidence interval. ref. = reference category

## Table S8. *Results of meta-analyzed model 3 per subgroup of studies based on depression assessment type.*

| **Depression assessment type** | **Outcome level** | **OR [95% CI]** |
| --- | --- | --- |
| Clinical interviews | Healthy controls | ref. |
|  | Depression only | 2.70 [2.53; 2.89] |
|  | Cardiometabolic disease only | 1.30 [1.19; 1.42] |
|  | Comorbidity | 2.90 [2.27; 3.70] |
| Self-report scales | Healthy controls | ref. |
|  | Depression only | 2.56 [1.47; 4.44] |
|  | Cardiometabolic disease only | 1.19 [1.01; 1.40] |
|  | Comorbidity | 3.16 [2.53; 3.96] |

*Abbreviations.* OR = Odds ratio. CI = confidence interval. ref. = reference category
